# The Pace of Change: Evaluating the Implementation of Age-Friendly Health Systems in Convenient Care Clinics

**DOI:** 10.1093/geroni/igaf122.501

**Published:** 2025-12-31

**Authors:** Grace Armstrong, Sarah Ball, Nicholas Schiltz, Brant Oliver, Anne Pohnert, Mary Dolansky

**Affiliations:** Case Western Reserve University, Cleveland, Ohio, United States; CVS Health, Woonsocket, Rhode Island, United States; Case Western Reserve University, Cleveland, Ohio, United States; Dartmouth Health, Hanover, New Hampshire, United States; CVS Health, Woonsocket, Rhode Island, United States; Case Western Reserve University, Cleveland, Ohio, United States

## Abstract

The Age-Friendly Health Systems 4Ms framework (What Matters, Mobility, Medication, Mentation) was implemented in CVS MinuteClinic convenient care clinics nationwide in May 2020. Since then, a vast collection of strategies have been used to enhance provider delivery of the 4Ms. These include documentation optimization, additional time for patient’s 65 years and up appointments, incorporating the 4Ms into annual provider performance reviews, and extensive education initiatives. These strategies can now be retrospectively examined to determine which had the greatest impact on the pace of organizational change. Statistical Process Control (SPC) analyses assessed performance variation across over 1,000,000 eligible visits from May 2021 to March 2025. Outcome measures included the proportion of eligible patients meeting each M of the 4M criteria, the proportion meeting all 4Ms criteria, and a composite score for 4M completion (M Score). Improvement was observed across all core measures: (1) “Matters” improved from 23.1% to 55.0%; (2) “Mobility” improved from 11.5% to 52.4%; (3) “Medication” improved from 18.7% to 50.1%; (4) “Mentation” improved from 6.4% to 47.9%; and (5) “4M” improved from 4.8% to 42.1%. “Average M Score” (Min-Max Range: 0, 4.0) performance also steadily improved from 0.61 at baseline to 2.05 in 2025. Overall, consistent performance improvements were observed from 2021 to 2025. The addition of time to the visit for patients ages 65 and up, creation and enhancement of provider Best Practice Alerts and the inclusion of 4Ms metrics in annual performance reviews were some of the strategies that significantly increased delivery of 4Ms care.

